# Progress and change

**DOI:** 10.1093/emph/eox003

**Published:** 2017-02-11

**Authors:** Charles L. Nunn, Stephen C. Stearns

**Affiliations:** a Duke University; b Yale University

We are happy to announce the transition in the Editor-in-Chief of *Evolution, Medicine and Public Health* (EMPH) from Stephen Stearns to Charles Nunn. It occurred on 1 January 2017. As part of this transition, we anticipate some turnover in the editorial board, for it coincides with the terms of our first group of Associate Editors. We thank all members of the editorial board for helping to grow the journal and for ensuring a rigorous review process that quickly delivers high-quality feedback to authors of submitted manuscripts. We also thank the many authors for submitting their best work to EMPH, and the referees for their constructive reviews. Finally, we thank the editorial and production team at Oxford University Press (OUP) for their efforts to keep us all moving forward in our service to the journal and the academic community, and for keeping the EMPH website populated with fresh and professionally formatted papers.

OUP waived publication fees from 2012 through 2015. When we began to charge publishing fees in 2016, we saw a predictable decline in submissions, yet we continued to attract an outstanding set of manuscripts for consideration. In addition, our acceptance rate in 2016 held steady at about 56%. All of the accepted papers are Open Access and thus available free-of-charge on the EMPH website.

We were also happy to see an impressively diverse set of Research Articles, Reviews, Commentaries, and Clinical Briefs published in 2016. The areas of medical science in which evolutionary thinking is already strong—cancer, antibiotic resistance and the hygiene hypothesis—are all areas that we would like to see better represented in the journal. However, their relative scarcity is actually testament to the success of evolutionary thinking in those areas—reviewers and editors at more established journals now accept the importance of evolutionary perspectives in these areas, leading to fewer submissions to EMPH. Thus, our inability to attract the best papers in some areas is actually a signal that the broader purpose of EMPH and the International Society for Evolution, Medicine and Public Health (ISEPMH) is succeeding.

Nunn’s goals as Editor-in-Chief are to increase the number of submissions while maintaining the high quality of articles accepted for publication, and to ensure that EMPH continues to cover the breadth of topics in evolutionary medicine. Working with OUP, we shall continue offering a world-class publishing venue—i.e. constructive reviews delivered quickly, with efficient movement of accepted papers into publication by the production department. EMPH is emerging as the “go-to” journal for top-notch research in evolutionary medicine, with an international following. To continue this growth, it is important for members of ISEMPH to submit their best work to EMPH, to participate actively as reviewers, to engage with our published articles and to cite them in forthcoming papers. Good science properly applied reduces suffering and saves lives. That’s what we want to publish.

